# Supplementary material for: Evolutionary Trends in the Mitochondrial Genome of Archaeplastida: How Does the GC Bias Affect the Transition from Water to Land?
Source: Plants (Basel). 2020 Mar 12;9(3):358. doi: 10.3390/plants9030358 (PMC7154891; doi:10.3390/plants9030358)
Supplement: Supplementary file 1 [file plants-09-00358-s001.pdf]

Supplementary Materials

Table S1. Genomic variables studied: Species, Genbank accession number, clade, genome length (GL), GC content (%GC), non-coding DNA (%NC), gene number (GN), number of protein-coding genes (NPG), number of repeated sequences (NRS) and repeated sequences total length (RSL).

Species	Genbank	Clade	GL	%GC	%NC	GN	NPG	NRS	RSL
Rickettsia prowazekii	NC_000963	Bacteria	1111523	29	25	888	835	-	-
Reclinomonas americana	NC_001823	Excavata	69034	26	20	97	67	-	-
Glaucocystis nostochinearum	NC_015117	Glaucophyta	34087	25	26	62	34	6	136
Cyanophora paradoxa	HQ849544	Glaucophyta	51557	26	34	77	44	117	6965
Cyanidioschyzon merolae	NC_000887	Rhodophyta	32211	27	24	62	34	9	233
Porphyra purpurea	NC_002007	Rhodophyta	36753	33	30	57	31	24	1055
Chondrus crispus	NC_001677	Rhodophyta	25836	27	29	57	29	12	400
Gracilariopsis andersonii	NC014772	Rhodophyta	27036	27	33	47	27	8	214
Ostreococcus tauri	NC_008290	Prasinophyta	44237	38	32	78	43	8	9948
Nephroselmis olivacea	NC_008239	Prasinophyta	45223	32	38	70	40	12	298
Micromonas sp.	NC_012643	Prasinophyta	47425	34	39	79	39	11	12482
Pycnococcus provasolii	NC_013935	Prasinophyta	24321	37	36	36	18	12	487
Oltmannsiellopsis viridis	NC_008256	Basal Chlorophyta	56761	33	47	63	36	514	16853
Pseudendoclonium akinetum	NC_005926	Basal Chlorophyta	95880	39	51	94	72	4386	154212
Prototheca wickerhamii	NC_001613	Basal Chlorophyta	55328	25	48	63	36	272	9050
Helicosporidium sp.	GQ339576	Basal Chlorophyta	37454	26	23	54	26	38	993
Scenedesmus obliquus	NC_002254	Chlorophyceae	42781	36	62	53	20	2524	89372
Pedinomonas minor	NC_000892	Basal Chlorophyta	25137	22	59	23	11	624	47999
Polytomella sp.	NC_013472	Chlorophyceae	13004	41	42	19	7	5	2501
Polytomella capuana	NC_010357	Chlorophyceae	12998	57	39	21	7	13	2165
Chlamydomonas eugametos	NC_001872	Chlorophyceae	22897	34	46	20	14	102	4040
Chlamydomonas reinhardtii	NC_001638	Chlorophyceae	15758	45	42	25	8	7	1056
Dunaliella salina	NC_012930	Chlorophyceae	28331	34	67	21	9	247	15401
Mesostigma viride	NC_008240	Basal Streptophyta	42424	32	35	70	41	6	151
Chlorokybus atmophyticus	NC_009630	Basal Streptophyta	201763	39	77	89	58	4293	126489
Chaetosphaeridium globosum	NC_004118	Basal Streptophyta	56574	34	45	77	46	13	557
Chara vulgaris	NC_005255	Charophyceae	67737	40	42	76	46 6	53	165
Nitella hyalina	NC_017598	Charophyceae	80192	38	42	77	43	39	1126
Marchantia polymorpha	NC_001660	Basal Embryophyta	186609	42	67	110	76	534	15900
Pleurozia purpurea	NC_013444	Basal Embryophyta	168526	45	75	101	69	493	13876

Megaceros aenigmaticus	NC_012651	Basal Embryophyta	184908	46	86	80	48	341	19022
Phaeoceros laevis	NC_013765	Basal Embryophyta	209482	44	89	73	38	134	5669
Physcomitrella patens	NC_007945	Basal Embryophyta	105340	40	67	69	42	145	3531
Cycas taitungensis	NC_010303	Spermatophyta	414903	46	92	70	39	101315	4210184
Zea perennis	NC_008331	Spermatophyta	570354	43	95	59	32	1311	102398
Carica papaya	NC_012116	Spermatophyta	476890	45	93	61	39	416	25220
Silene latifolia	NC_014487	Spermatophyta	253413	42	89	48	30	376	32474

Table S2. Presence of mitochondrial genes (1). Number of genes/species and number of studied species/gene are given .

Gene	Functional category	Gen type	Functional category	Gen type	Especies/gen	*Rickettsia_prowazekii*	*Reclinomonas americana*	*Homo_sapiens*	*Glaucocystis nostochinearum*	*Cyanophora paradoxa*	*Gracilariopsis andersonii*	*Chondrus_crispus*	*Porphyra_purpurea*	*Cyanidioschyzon_merolae*	*Mesostigma_viride*	*Chlorokybus_atmophyticus*	*Nitella hyalina*	*Chara_vulgaris*	*Chaetosphaeridium globosum*	*Pleurozia_purpurea*	*Marchantia_polymorpha*	*Nothoceros_aenigmaticus*	*Phaeoceros_laevis*	*Physcomitrella_patens*	*Cycas_taitungensis*	*Zea_perennis*	*Carica_papaya*	*Silene_latifolia*	*Pycnococcus_provasolii*	*Micromonas_sp.*	*Nephroselmis_olivacea*	*Ostreococcus_tauri*	*Pseudendoclonium_akinetum*	*Oltmannsiellopsis_viridis*	*Helicosporidium_sp.*	*Prototheca_wickerhamii*	*Pedinomonas_minor*	*Scenedesmus_obliquus*	*Chlamydomonas_reinhardtii*	*Polytomella_sp.*	*Dunaliella salina*	*Chlamydomonas_moewusii*	*Polytomella capuana*
						81	85	33	57	60	40	49	47	54	60	69	66	66	65	63	75	50	46	62	61	49	56	41	34	53	62	62	60	52	56	56	22	34	21	20	20	14	9
MTTB	Protein import and maturation	Celu	N	1	24		1					1	1		1	1	1	1	1	1	1	1	1	1	1	1	1	1		1	1	1	1	1	1	1							
SECY	Protein import and maturation	Celu	N	1	3	1	1				1																																
YEJU	Protein import and maturation	Celu	O	1	12	1	1							1			1	1		1	1			1	1	1	1	1															
YEJV	Protein import and maturation	Celu	O	1	11	1	1										1	1		1	1			1	1	1	1	1															

YEJR	Protein import and maturation	Celu	O	1	7	1	1						1			1	1			1			1													
CCMFN	Protein import and maturation	Celu	O	1	6														1				1	1	1	1	1									
CCMFC	Protein import and maturation	Celu	O	1	5														1					1	1	1	1									
PHOR	two components histidin-kinase sensor	Celu	T	1	2	1																1														
RPS3	Ribosomal proteins	Info	J	2	28	1	1		1	1	1	1	1	1	1	1	1	1	1	1	1			1	1	1	1	1	1	1	1	1	1	1	1	1
RPS12	Ribosomal proteins	Info	J	2	27	1	1		1	1	1	1	1	1	1	1	1	1	1	1	1			1	1	1	1		1	1	1	1	1	1	1	1
RPL16	Ribosomal proteins	Info	J	2	26	1	1		1	1	1	1	1	1	1	1	1	1	1	1	1			1	1	1	1			1	1	1	1	1	1	1
RPS11	Ribosomal proteins	Info	J	2	24	1	1		1	1	1	1	1	1	1	1	1	1	1	1	1			1	1					1	1	1	1	1	1	1
RPS4	Ribosomal	Info	J	2	23	1	1			1				1	1	1	1	1	1	1	1			1	1	1	1	1	1	1	1	1	1		1	1

	proteins																																				
RPL5	Ribosomal proteins	Info	J	2	22	1	1		1	1				1	1	1	1	1	1	1	1			1	1		1	1		1	1	1	1		1	1	
RPS13	Ribosomal proteins	Info	J	2	22	1	1		1	1					1	1			1	1	1	1	1	1	1	1	1			1	1	1	1	1	1	1	
RPS14	Ribosomal proteins	Info	J	2	22	1	1							1	1	1	1	1	1	1	1	1		1	1		1	1		1	1	1	1	1	1	1	
RPS19	Ribosomal proteins	Info	J	2	21	1	1		1	1					1	1	1	1	1	1	1			1	1		1			1	1	1	1	1	1	1	
RPS7	Ribosomal proteins	Info	J	2	20	1	1		1	1					1	1	1	1	1	1	1			1	1	1	1			1	1	1			1	1	
RPS10	Ribosomal proteins	Info	J	2	19	1	1		1	1					1	1	1	1	1	1	1				1		1			1	1	1	1		1	1	
RPS2	Ribosomal proteins	Info	J	2	19	1	1								1	1	1	1	1	1	1			1	1	1				1	1	1	1	1	1	1	
RPL6	Ribosomal proteins	Info	J	2	18	1	1		1	1				1	1	1	1	1	1	1	1			1						1	1	1			1	1	
RNL	ribosomal RNAs	Info	J	2	17		1		1	1	1	1	1		1	1	1	1	1										1		1		1	1	1	1	
RNS	ribosomal RNAs	Info	J	2	15		1		1	1			1		1	1	1	1	1										1		1		1	1	1		1
RPL2	Ribosomal	Info	J	2	13	1	1		1	1						1	1	1	1	1	1			1	1		1										

	proteins																																									
RPS 1	Ribosomal proteins	Info	J	2	13	1	1								1	1	1	1	1	1	1			1	1	1	1															
RPL 14	Ribosomal proteins	Info	J	2	12	1	1			1				1	1	1	1	1												1	1	1	1									
RPS 8	Ribosomal proteins	Info	J	2	9	1	1							1		1				1	1									1	1	1										
RPL 20	Ribosomal proteins	Info	J	2	4	1	1				1			1																												
RN PB	Catalytic subunit of Rnase	Info	J	2	3		1																								1	1										
RN L_A	ribosomal RNAs	Info	J	2	2																																1					1
RN L_B	ribosomal RNAs	Info	J	2	2																																1					1
RPL 1	Ribosomal proteins	Info	J	2	2	1	1																																			
RPL 10	Ribosomal proteins	Info	J	2	2	1	1																																			
RPL 11	Ribosomal proteins	Info	J	2	2	1	1																																			
RPL 18	Ribosomal	Info	J	2	2	1	1																																			

	proteins																																										
RPL 19	Ribosomal proteins	Info	J	2	2	1	1																																				
RPL 27	Ribosomal proteins	Info	J	2	2	1	1																																				
RPL 31	Ribosomal proteins	Info	J	2	2	1	1																																				
RPL 32	Ribosomal proteins	Info	J	2	2	1	1																																				
RPL 34	Ribosomal proteins	Info	J	2	2	1	1																																				
RPS 18	Ribosomal proteins	Info	J	2	2	1															1																						
RPOA	Transcription	Info	K	2	2	1	1																																				
RPOB	Transcription	Info	K	2	2	1	1																																				
RPOC	Transcription	Info	K	2	2	1	1																																				
RPOD	Transcription	Info	K	2	2	1	1																																				
MATR	Maturase-related protein	Info	L	2	4																									1	1	1	1															
RTL	Reverse transcriptase-	Info	L	2	3								1							1																			1				

	like protein																																										
DPO	DNA polymerase gene	Info	L	2	2								1															1															
COB	Respiration and oxidative phosphorylation	Mtbs	C	3	38	1	1	1	1	1	1	1	1	1	1	1	1	1	1	1	1	1	1	1	1	1	1	1	1	1	1	1	1	1	1	1	1	1	1	1	1	1	1
COX1	Respiration and oxidative phosphorylation	Mtbs	C	3	38	1	1	1	1	1	1	1	1	1	1	1	1	1	1	1	1	1	1	1	1	1	1	1	1	1	1	1	1	1	1	1	1	1	1	1	1	1	1
NAD5	Respiration and oxidative phosphorylation	Mtbs	C	3	38	1	1	1	1	1	1	1	1	1	1	1	1	1	1	1	1	1	1	1	1	1	1	1	1	1	1	1	1	1	1	1	1	1	1	1	1	1	1
NAD6	Respiration and oxidative phosphorylation	Mtbs	C	3	38	1	1	1	1	1	1	1	1	1	1	1	1	1	1	1	1	1	1	1	1	1	1	1	1	1	1	1	1	1	1	1	1	1	1	1	1	1	1
ATP6	Respiration and oxidati	Mtbs	C	3	33	1	1	1	1	1	1	1	1	1	1	1	1	1	1	1	1	1	1	1	1	1	1	1	1	1	1	1	1	1	1	1	1	1					

	ve phosp horyla tion																																					
CO X2	Respir ation and oxidati ve phosp horyla tion	M tb s	C	3	32	1	1	1	1	1	1	1	1	1	1	1	1	1	1	1	1	1	1	1	1	1	1	1	1	1	1	1	1	1	1	1		1
CO X3	Respir ation and oxidati ve phosp horyla tion	M tb s	C	3	32	1	1	1	1	1	1	1	1	1	1	1	1	1	1	1	1	1	1	1	1	1	1	1	1	1	1	1	1	1	1	1		1
ATP 8	Respir ation and oxidati ve phosp horyla tion	M tb s	C	3	30	1	1	1	1	1	1	1	1	1	1	1	1	1	1	1	1			1	1	1	1	1	1	1	1	1	1	1	1	1	1	
ATP 9	Respir ation and oxidati ve phosp horyla tion	M tb s	C	3	30		1		1	1	1	1	1	1	1	1	1	1	1	1	1	1	1	1	1	1	1	1	1	1	1	1	1	1	1	1		1
ATP 4	Respir ation and oxidati ve phosp	M tb s	C	3	27		1			1		1	1	1	1	1	1	1	1	1	1	1	1	1	1	1	1	1	1	1	1	1	1	1	1	1		

	horylation																																			
ATP1	Respiration and oxidative phosphorylation	Mtbs	C	3	22	1	1								1	1	1	1	1	1	1	1	1	1	1	1	1	1			1	1	1	1	1	1
SDH3	Respiration and oxidative phosphorylation	Mtbs	C	3	19	1	1		1	1	1	1	1	1	1	1	1	1	1	1	1	1		1	1		1									
SDH4	Respiration and oxidative phosphorylation	Mtbs	C	3	19	1	1		1	1	1	1	1	1	1	1	1	1	1	1	1	1	1	1			1									
SDH2	Respiration and oxidative phosphorylation	Mtbs	C	3	10	1	1				1	1	1	1					1			1	1											1		
ATP4_2	Respiration and oxidative phosphorylation	Mtbs	C	3	2																									1		1				

ATP6_2	Respiration and oxidative phosphorylation	Mtbs	C	3	2																					1				1													
ATP8_2	Respiration and oxidative phosphorylation	Mtbs	C	3	2																									1		1											
COX1_2	Respiration and oxidative phosphorylation	Mtbs	C	3	2																									1		1											
COX11	Protein import and maturation	Mtbs	C	3	2	1	1																																				
NAD1	Respiration and oxidative phosphorylation	Mtbs	H	3	38	1	1	1	1	1	1	1	1	1	1	1	1	1	1	1	1	1	1	1	1	1	1	1	1	1	1	1	1	1	1	1	1	1	1	1	1	1	1
NAD2	Respiration and oxidative	Mtbs	H	3	38	1	1	1	1	1	1	1	1	1	1	1	1	1	1	1	1	1	1	1	1	1	1	1	1	1	1	1	1	1	1	1	1	1	1	1	1	1	1

	phosphorylation																																										
NAD4	Respiration and oxidative phosphorylation	Mtbs	H	3	38	1	1	1	1	1	1	1	1	1	1	1	1	1	1	1	1	1	1	1	1	1	1	1	1	1	1	1	1	1	1	1	1	1	1	1	1	1	1
NAD3	Respiration and oxidative phosphorylation	Mtbs	H	3	33	1	1	1	1	1	1	1	1	1	1	1	1	1	1	1	1	1	1	1	1	1	1	1	1	1	1	1	1	1	1	1	1	1					
NAD4L	Respiration and oxidative phosphorylation	Mtbs	H	3	33	1	1	1	1	1	1	1	1	1	1	1	1	1	1	1	1	1	1	1	1	1	1	1	1	1	1	1	1	1	1	1	1	1					
NAD9	Respiration and oxidative phosphorylation	Mtbs	H	3	24	1	1		1	1					1	1	1	1	1	1	1	1	1	1	1	1	1	1		1	1	1		1	1	1							
NAD7	Respiration and oxidative phosphorylation	Mtbs	H	3	22	1	1		1	1					1	1	1	1	1		1			1	1	1	1	1		1	1	1	1	1	1	1							

NAD10	Respiration and oxidative phosphorylation	Mtbs	H	3	4		1									1															1	1	
NAD11	Respiration and oxidative phosphorylation	Mtbs	H	3	3		1		1	1																							
YEJW	Protein import and maturation	Mtbs	Q	3	3	1	1							1																			
ORF103	Poo	Poo	S	6	3											1						1	1										
ORF109	Poo	Poo	S	6	3																1	1	1										
ORF110	Poo	Poo	S	6	3											1						1	1										
ORF137	Poo	Poo	S	6	3							1									1	1											
ORF61	Poo	Poo	S	6	3						1										1												1
ORF760	Poo	Poo	S	6	3												1	1							1								
ORF101	Poo	Poo	S	6	2														1														1

ORF 103_2	Poo	Poo	S	6	2																1	1																			
ORF 108	Poo	Poo	S	6	2										1																	1									
ORF 118	Poo	Poo	S	6	2																1																				1
ORF 126	Poo	Poo	S	6	2													1			1																				
ORF 134	Poo	Poo	S	6	2																1	1																			
ORF 150	Poo	Poo	S	6	2										1																										1
ORF 151	Poo	Poo	S	6	2																																1			1	
ORF 162	Poo	Poo	S	6	2				1					1																											
ORF 164	Poo	Poo	S	6	2			1																						1											
ORF 166	Poo	Poo	S	6	2				1																									1							
ORF 169	Poo	Poo	S	6	2	1														1																					
ORF 170	Poo	Poo	S	6	2										1			1																							
ORF 174	Poo	Poo	S	6	2									1																					1						
ORF 196	Poo	Poo	S	6	2															1	1																				

ORF 207	Poo	Poo	S	6	2								1	1				
ORF 220	Poo	Poo	S	6	2					1						1		
ORF 228	Poo	Poo	S	6	2									1		1		
ORF 260	Poo	Poo	S	6	2					1								1
ORF 267	Poo	Poo	S	6	2				1				1					
ORF 448	Poo	Poo	S	6	2						1	1						
ORF 533	Poo	Poo	S	6	2	1									1			
ORF 544	Poo	Poo	S	6	2	1		1										
ORF 558	Poo	Poo	S	6	2						1	1						
ORF 576	Poo	Poo	S	6	2						1	1						
ORF 60	Poo	Poo	S	6	2		1							1				
ORF 63	Poo	Poo	S	6	2									1			1	
ORF 69	Poo	Poo	S	6	2									1			1	
ORF 70	Poo	Poo	S	6	2												1	1

ORF 74	Poo	Poo	S	6	2							1									1																				
ORF 85	Poo	Poo	S	6	2							1																					1								
ORF 90	Poo	Poo	S	6	2																												1					1			
ORF 99	Poo	Poo	S	6	2					1											1																				
ORF 211	Poo	Poo	S	6	1				1																																
ORF 251	Poo	Poo	S	6	1				1																																
ORF 287	Poo	Poo	S	6	1				1																																
RR N5	riboso mal RNAs	RNA	RIB	4	24		1		1	1		1		1	1	1	1	1	1	1	1	1	1	1	1	1	1	1			1	1		1	1	1					
RR N18	riboso mal RNAs	RNA	RIB	4	10							1								1		1	1	1	1	1	1	1								1					
RR N26	riboso mal RNAs	RNA	RIB	4	9															1	1	1	1	1	1	1	1	1													
RRL	riboso mal RNAs	RNA	RIB	4	3	1																								1		1									
RR NL1	riboso mal RNAs	RNA	RIB	4	3																																		1	1	1
RR NL4	riboso mal RNAs	RNA	RIB	4	3																																		1	1	1
RR NL5	riboso mal RNAs	RNA	RIB	4	3																																		1	1	1

RRNL6	ribosomal RNAs	RNA	RIB	4	3																																		1	1	1		
RRNS1	ribosomal RNAs	RNA	RIB	4	3																																		1	1	1		
RRNS2	ribosomal RNAs	RNA	RIB	4	3																																		1	1	1		
RRNS3	ribosomal RNAs	RNA	RIB	4	3																																		1	1	1		
RRS	ribosomal RNAs	RNA	RIB	4	3	1					1																				1												
RRNL	ribosomal RNAs	RNA	RIB	4	2									1																													1
RRNL2	ribosomal RNAs	RNA	RIB	4	2																																			1	1		
RRNL3	ribosomal RNAs	RNA	RIB	4	2																																			1	1		
RRNL7	ribosomal RNAs	RNA	RIB	4	2																																		1	1			
RRNL8	ribosomal RNAs	RNA	RIB	4	2																																		1	1			
RRNS4	ribosomal RNAs	RNA	RIB	4	2																																		1	1			
TRNA-MET	Transfer RNAs	RNA	Tran	5	36	1	1	1	1	1	1	1	1	1	1	1	1	1	1	1	1	1	1	1	1	1	1	1	1		1	1	1	1	1	1		1	1	1	1	1	1
TRNA-GLN	Transfer RNAs	RNA	Tran	5	35	1	1	1	1	1	1	1	1	1	1	1	1	1	1	1	1	1	1	1	1	1	1		1	1	1	1	1	1	1	1	1	1	1		1	1	

TRNA-CYS	Transfer RNAs	RNA	Tran	5	33	1	1	1	1	1	1	1	1	1	1	1	1	1	1	1	1	1	1	1	1	1	1	1	1	1	1	1	1	1	1	1	1	1				
TRNA-GLU	Transfer RNAs	RNA	Tran	5	33	1	1	1	1	1	1	1	1	1	1	1	1	1	1	1	1	1	1	1	1	1	1	1	1	1	1	1	1	1	1	1	1	1				
TRNA-TRP	Transfer RNAs	RNA	Tran	5	33	1	1	1	1	1		1	1	1	1	1	1	1	1	1	1	1	1	1	1		1	1	1		1	1	1	1	1	1	1	1	1		1	1
TRNA-HIS	Transfer RNAs	RNA	Tran	5	32	1	1	1	1	1		1	1	1	1	1	1	1	1	1	1	1	1	1	1	1	1	1	1	1	1	1	1	1	1	1	1	1				
TRNA-PRO	Transfer RNAs	RNA	Tran	5	32	1	1	1	1	1	1	1	1	1	1	1	1	1	1	1	1	1	1	1	1	1	1	1	1	1	1	1	1	1	1	1		1				
TRNA-TYR	Transfer RNAs	RNA	Tran	5	32	1	1	1	1	1		1	1	1	1	1	1	1	1	1	1	1	1	1	1	1	1	1	1	1	1	1	1	1	1	1	1	1				
TRNA-PHE	Transfer RNAs	RNA	Tran	5	31	1	1	1	1	1	1	1	1	1	1	1	1	1	1	1	1	1	1	1	1	1	1			1	1	1	1	1	1	1	1	1				
TRNA-ASP	Transfer RNAs	RNA	Tran	5	30	1	1	1	1	1		1	1	1	1	1	1	1	1	1	1	1	1	1	1	1	1		1	1	1	1	1	1	1	1		1				
TRNA-LEU	Transfer RNAs	RNA	Tran	5	30	1	1	1	1	1	1	1	1	1	1	1	1	1	1	1	1	1	1	1	1				1	1	1	1	1	1	1	1	1	1				
TRNA-ASN	Transfer RNAs	RNA	Tran	5	29	1	1	1	1	1	1	1	1	1	1	1	1	1	1	1	1				1	1	1	1	1	1	1	1	1	1	1	1		1				
TRNA-GLY	Transfer RNAs	RNA	Tr	5	29	1	1	1	1	1	1	1	1	1	1	1	1	1	1	1	1	1	1	1	1		1			1	1	1	1	1	1	1		1				

			an																																								
TRNA-ILE	Transfer RNAs	RNA	Tran	5	29	1	1	1	1	1		1	1	1	1	1	1	1	1	1		1	1	1	1	1	1	1	1		1	1	1	1	1	1		1					
TRNA-LYS	Transfer RNAs	RNA	Tran	5	29	1	1	1	1	1	1	1	1	1	1	1	1	1	1	1	1	1	1	1	1	1	1				1	1	1	1	1	1		1					
TRNA-SER	Transfer RNAs	RNA	Tran	5	29	1	1	1	1	1	1	1	1	1	1	1	1	1	1	1	1			1	1	1	1		1	1	1	1	1	1	1	1		1					
TRNA-VAL	Transfer RNAs	RNA	Tran	5	28	1	1	1	1	1	1	1	1	1	1	1	1	1	1	1	1		1	1	1				1	1	1	1	1	1	1	1		1					
TRNA-ALA	Transfer RNAs	RNA	Tran	5	27	1	1	1	1	1	1	1	1	1	1	1	1	1	1	1	1	1	1	1						1	1	1	1	1	1	1		1					
TRNA-ARG	Transfer RNAs	RNA	Tran	5	25	1	1	1	1	1	1	1	1	1	1	1	1	1	1	1	1			1	1						1	1	1	1	1	1		1					
TRNA-THR	Transfer RNAs	RNA	Tran	5	17	1		1	1							1	1	1	1		1	1	1	1						1	1	1	1		1	1							
TRNA-FMET	Transfer RNAs	RNA	Tran	5	2	1															1																						
				Celu		5	5	0	0	0	1	1	1	2	1	1	4	4	1	5	4	1	2	5	5	5	5	5	0	1	1	1	1	1	1	1	0	0	0	0	0	0	0
				Info		32	34	0	13	15	6	5	8	11	17	19	17	17	17	17	17	2	1	14	15	9	13	6	5	15	18	16	14	10	15	14	3	0	1	0	0	2	0

	Mtbs	21	25	13	19	20	17	18	18	19	20	21	20	20	21	19	20	19	18	20	19	19	20	18	15	21	19	22	17	19	18	18	11	13	7	7	7	7	7
	rRNA	2	1	0	1	1	1	2	0	2	1	1	1	1	1	3	2	3	3	3	3	3	3	3	0	1	1	3	0	1	1	2	0	0	10	12	9	0	1
	tRNA	21	19	20	20	19	14	19	19	19	19	20	20	20	20	19	20	16	17	19	18	13	15	9	14	15	20	20	20	19	20	20	8	19	3	1	3	3	1
	Poo	0	1	0	4	5	1	4	1	1	2	7	4	4	5	0	12	9	5	1	1	0	0	0	0	0	3	0	8	2	1	1	0	2	0	0	1	2	0

Table S3. GC content in the ribosomal subunits (RSU) from the 100 species of Streptophyta used in the analysis at each subunit. List of species used to estimate the phylogeny of each ribosomal subunit.

RSU	Clade	Species	%GC
mtssu	Angiosperms	*Ascarina_rubricauli*	52.05
mtssu	Gimnosperms	*Abies_homolepis*	51.83
mtssu	Angiosperms	*Acorus_calamus*	52.65
mtssu	Monilophyta	*Adiantum_pedatum*	47.84
mtssu	Angiosperms	*Alisma_plantago-aqua*	51.81
mtssu	Angiosperms	*Amborella_trichopoda*	51.19
mtssu	Monilophyta	*Angyopteris_lygodifo*	48.03
mtssu	Angiosperms	*Aristolochia_macroph*	51.14
mtssu	Angiosperms	*Artemisia_annua*	48.26
mtssu	Angiosperms	*Asarum_canadense*	51.29
mtssu	Angiosperms	*Asparagus_officinali*	52.04
mtssu	Monilophyta	*Asplenium_nidus*	49.02
mtssu	Angiosperms	*Austrobaileya_scande*	51.63
mtssu	Monilophyta	*Azolla_pinnata*	48.96
mtssu	Angiosperms	*Berberis_bealei*	52.25
mtssu	Angiosperms	*Beta_vulgaris*	51.49
mtssu	Monilophyta	*Botrychium_dissectum*	48.16
mtssu	Angiosperms	*Brasenia_schreberi*	52.00
mtssu	Angiosperms	*Brassica_rapa*	51.36
mtssu	Angiosperms	*Buxus_sp*	51.63
mtssu	Marchantiophyta	*Calypogeia_muelleria*	47.90
mtssu	Angiosperms	*Canella_winterana*	51.55
mtssu	Angiosperms	*Carica_papaya*	51.07
mtssu	Angiosperms	*Ceratophyllum_demers*	52.15
mtssu	Coleochaetophyta	*Chaetosphaeridium_gl*	45.24
mtssu	Charophyta	*Chara_sp*	49.20
mtssu	Charophyta	*Chara_vulgaris*	50.11
mtssu	Angiosperms	*Citrullus_lanatus*	51.96
mtssu	Angiosperms	*Crossosoma_bigelovii*	51.27
mtssu	Gimnosperms	*Cycas_revoluta*	49.85
mtssu	Gimnosperms	*Cycas_taitungensis*	50.56
mtssu	Angiosperms	*Cytinus_ruber*	52.08
mtssu	Angiosperms	*Digitalis_purpurea*	52.49
mtssu	Gimnosperms	*Dioon_edule*	51.16
mtssu	Angiosperms	*Dioscorea_sp*	51.94
mtssu	Lycopodiophyta	*Diphasiastrum_digita*	49.09
mtssu	Monilophyta	*Diplazium_pycnocarpo*	48.87
mtssu	Angiosperms	*Doryphora_sassafras*	51.64
mtssu	Monilophyta	*Equisetum_arvense*	48.00
mtssu	Angiosperms	*Erodium_chrysanthum*	51.20
mtssu	Angiosperms	*Euptelea_polyandra*	51.69
mtssu	Angiosperms	*Geranium_himalayense*	50.95
mtssu	Gimnosperms	*Ginkgo_biloba*	50.80
mtssu	Angiosperms	*Grevillea_robusta*	51.83
mtssu	Angiosperms	*Hedyosmum_arborescen*	51.56
mtssu	Angiosperms	*Helosis_cayennensis*	52.04
mtssu	Lycopodiophyta	*Huperzia_lucidula*	49.09
mtssu	Lycopodiophyta	*Isoetes_engelmanii*	49.70
mtssu	Lycopodiophyta	*Isoetes_histrix*	49.85
mtssu	Lycopodiophyta	*Isoetes_taiwanensis*	49.27
mtssu	Angiosperms	*Lactuca_sativa*	51.17
mtssu	Angiosperms	*Liriodendron_tulipif*	51.28

mtssu	Angiosperms	*Magnolia_grandiflora*	51.49
mtssu	Marchantiophyta	*Marchantia_plymorpha*	47.39
mtssu	Angiosperms	*Medicago_truncatula*	51.06
mtssu	Anthocerotophyta	*Megaceros_tosanus*	46.42
mtssu	Mesostigmatophyta	*Mesostigma_viride*	48.65
mtssu	Angiosperms	*Monsonia_emarginata*	51.01
mtssu	Gimnosperms	*Nageia_nagi*	52.05
mtssu	Angiosperms	*Nelumbo_nucifera*	51.69
mtssu	Angiosperms	*Nicotiana_tabacum*	51.56
mtssu	Charophyta	*Nitella_hyalina*	47.85
mtssu	Anthocerotophyta	*Nothoceros_aenigmati*	45.81
mtssu	Anthocerotophyta	*Notothylas_orbicular*	46.07
mtssu	Angiosperms	*Nymphaea_sp*	51.89
mtssu	Monilophyta	*Ophioglossum_vulgatu*	49.60
mtssu	Angiosperms	*Oryza_sativa*	44.41
mtssu	Angiosperms	*Panax_ginseng*	51.54
mtssu	Angiosperms	*Petrophile_canescens*	51.83
mtssu	Anthocerotophyta	*Phaeoceros_laevis*	46.97
mtssu	Monilophyta	*Phegopteris_hexagono*	49.11
mtssu	Bryophyta	*Physcomitrella_paten*	46.68
mtssu	Gimnosperms	*Pinus_sp*	51.53
mtssu	Gimnosperms	*Pinus_strobus*	50.82
mtssu	Angiosperms	*Platanus_occidentali*	51.83
mtssu	Marchantiophyta	*Pleurozia_purpurea*	47.83
mtssu	Gimnosperms	*Podocarpus_costarice*	51.85
mtssu	Angiosperms	*Podophyllum_peltatum*	50.99
mtssu	Monilophyta	*Polypodium_aureum*	48.31
mtssu	Gimnosperms	*Pseudotsuga_menziesi*	49.99
mtssu	Angiosperms	*Riccinus_communis*	51.72
mtssu	Angiosperms	*Sambucus_canadensis*	51.56
mtssu	Angiosperms	*Saruma_henryi*	51.33
mtssu	Angiosperms	*Schisandra_sphenanth*	51.64
mtssu	Angiosperms	*Silene_latifolia*	52.90
mtssu	Angiosperms	*Solanum_tuberosum*	49.85
mtssu	Angiosperms	*Spathiphyllum_wallis*	51.28
mtssu	Bryophyta	*Sphagnum_palustris*	46.84
mtssu	Bryophyta	*Syntrichia_ruralis*	47.32
mtssu	Bryophyta	*Takakia_lepidozioide*	48.14
mtssu	Angiosperms	*Tasmannia_insipida*	51.82
mtssu	Angiosperms	*Theobroma_cacao*	51.14
mtssu	Angiosperms	*Thottea_tomentosa*	50.85
mtssu	Angiosperms	*Tofieldia_calyculata*	51.29
mtssu	Angiosperms	*Triticum_aestivum*	51.69
mtssu	Angiosperms	*Vigna_radiata*	51.60
mtssu	Angiosperms	*Xanthosoma_mafaffa*	51.10
mtssu	Gimnosperms	*Zamia_integrifolia*	50.89
mtssu	Angiosperms	*Zea_luxurians*	51.82
mtssu	Klebsormidiophyta	*Chlorokybus_atmophyt*	49.16
mtlsu	Angiosperms	*Akebia_quinata*	59.31
mtlsu	Angiosperms	*Alisma_plantago-aqua*	54.95
mtlsu	Angiosperms	*Amborella_trichopoda*	58.86
mtlsu	Angiosperms	*Annona_muricata*	55.11
mtlsu	Anthocerotophyta	*Anthoceros_agrestis*	51.81
mtlsu	Angiosperms	*Atherosperma_moschat*	54.87
mtlsu	Bryophyta	*Atrichum_angustatum*	53.87
mtlsu	Marchantiophyta	*Bazzania_trilobata*	54.17

mtlsu	Marchantiophyta	*Blasia_pusilla*	53.62
mtlsu	Angiosperms	*Brasenia_schreberi*	53.59
mtlsu	Bryophyta	*Buxbaumia_aphylla*	53.87
mtlsu	Angiosperms	*Buxus_sempervirens*	56.95
mtlsu	Angiosperms	*Cabomba_sp*	54.35
mtlsu	Gimnosperms	*Cedrus_deodara*	56.16
mtlsu	Bryophyta	*Ceratodon_purpureus*	53.73
mtlsu	Angiosperms	*Ceratophyllum_demers*	53.71
mtlsu	Coleochaetophyta	*Chaetosphaeridium_gl*	51.40
mtlsu	Charophyta	*Chara_vulgaris*	54.72
mtlsu	Klebsormidiophyta	*Chlorokybus_atmophyt*	55.10
mtlsu	Marchantiophyta	*Conocephalum_conicum*	53.64
mtlsu	Angiosperms	*Croomia_pauciflora*	57.33
mtlsu	Gimnosperms	*Cycas_revoluta*	54.64
mtlsu	Gimnosperms	*Cycas_taitungensis*	54.19
mtlsu	Monilophyta	*Danaea_elliptica*	54.61
mtlsu	Angiosperms	*Degeneria_vitiensis*	54.82
mtlsu	Anthocerotophyta	*Dendroceros_granulat*	52.97
mtlsu	Bryophyta	*Dicranum_scoparium*	53.84
mtlsu	Angiosperms	*Didymeles_perrieri*	54.46
mtlsu	Bryophyta	*Diphyscium_foliosum*	54.19
mtlsu	Angiosperms	*Drimys_winteri*	56.15
mtlsu	Marchantiophyta	*Dumortiera_hirsuta*	53.75
mtlsu	Monilophyta	*Equisetum_hyemale*	54.55
mtlsu	Bryophyta	*Fissidens_dubius*	53.99
mtlsu	Bryophyta	*Fontinalis_antipyret*	54.06
mtlsu	Marchantiophyta	*Frullania_dilatata*	53.80
mtlsu	Gimnosperms	*Ginkgo_biloba*	54.37
mtlsu	Angiosperms	*Grevillea_robusta*	56.15
mtlsu	Angiosperms	*Hortonia_floribunda*	55.42
mtlsu	Angiosperms	*Houttuynia_cordata*	55.85
mtlsu	Bryophyta	*Hypnum_imponens*	54.32
mtlsu	Angiosperms	*Idiospermum_australi*	54.63
mtlsu	Lycopodiophyta	*Isoetes_malinvernian*	54.93
mtlsu	Angiosperms	*Kadsura_japonica*	54.69
mtlsu	Klebsormidiophyta	*Klebsormidium_flacci*	53.91
mtlsu	Marchantiophyta	*Leiomylia_anomala*	54.27
mtlsu	Marchantiophyta	*Lejeunea_cavifolia*	54.62
mtlsu	Marchantiophyta	*Lepidogyna_hodgsonia*	54.18
mtlsu	Angiosperms	*Liriodendron_chinens*	55.56
mtlsu	Marchantiophyta	*Lophocolea_heterophy*	54.30
mtlsu	Marchantiophyta	*Lophozia_gillmani*	54.61
mtlsu	Marchantiophyta	*Lunularia_cruciata*	53.90
mtlsu	Angiosperms	*Magnolia_tripetala*	55.56
mtlsu	Monilophyta	*Marattia_attenuata*	54.65
mtlsu	Marchantiophyta	*Marchantia_polymorph*	53.80
mtlsu	Marchantiophyta	*Marsupella_emarginat*	54.41
mtlsu	Angiosperms	*Medicago_truncatula*	55.30
mtlsu	Mesostigmatophyta	*Mesostigma_viride*	55.26
mtlsu	Angiosperms	*Myristica_fragrans*	56.36
mtlsu	Charophyta	*Nitella_hyalina*	54.94
mtlsu	Anthocerotophyta	*Nothoceros_aenigmati*	53.04
mtlsu	Anthocerotophyta	*Notothylas_breutelii*	52.69
mtlsu	Marchantiophyta	*Nowellia_curvifolia*	54.27
mtlsu	Marchantiophyta	*Odontoschisma_denuda*	54.22
mtlsu	Angiosperms	*Oenothera_berteroana*	54.75

mtlsu	Monilophyta	*Ophioglossum_lusitan*	55.43
mtlsu	Marchantiophyta	*Pallavicinia_lyellii*	54.51
mtlsu	Marchantiophyta	*Pellia_epiphylla*	54.50
mtlsu	Anthocerotophyta	*Phaeoceros_carolinia*	52.69
mtlsu	Anthocerotophyta	*Phaeoceros_laevis*	52.86
mtlsu	Gimnosperms	*Phyllocladus_aspleni*	55.50
mtlsu	Bryophyta	*Physcomitrella_paten*	54.22
mtlsu	Angiosperms	*Piper_betle*	57.84
mtlsu	Marchantiophyta	*Plagiochila_porelloi*	52.49
mtlsu	Angiosperms	*Platanus_occidentali*	57.53
mtlsu	Angiosperms	*Pleea_tenuifolia*	57.38
mtlsu	Marchantiophyta	*Pleurozia_purpurea*	54.47
mtlsu	Gimnosperms	*Podocarpus_macrophyl*	55.89
mtlsu	Bryophyta	*Polytrichum_juniperi*	53.46
mtlsu	Marchantiophyta	*Porella_pinnata*	54.07
mtlsu	Marchantiophyta	*Ptilidium_pulcherrim*	54.08
mtlsu	Marchantiophyta	*Radula_complanata*	53.59
mtlsu	Marchantiophyta	*Reboulia_hemisphaeri*	54.00
mtlsu	Marchantiophyta	*Riccia_sorocarpa*	53.70
mtlsu	Marchantiophyta	*Ricciocarpos_natans*	53.70
mtlsu	Angiosperms	*Saruma_henryi*	55.28
mtlsu	Angiosperms	*Saururus_cernuus*	55.64
mtlsu	Marchantiophyta	*Scapania_nemorea*	54.02
mtlsu	Angiosperms	*Schisandra_sphenanth*	54.71
mtlsu	Marchantiophyta	*Solenostoma_hyalinum*	54.04
mtlsu	Angiosperms	*Spathiphyllum_wallis*	54.28
mtlsu	Marchantiophyta	*Sphaerocarpos_donnel*	54.00
mtlsu	Bryophyta	*Sphagnum_capillifoli*	51.84
mtlsu	Bryophyta	*Sphagnum_recurvum*	52.60
mtlsu	Gimnosperms	*Stangeria_eriopus*	54.49
mtlsu	Marchantiophyta	*Symphyogyna_circinat*	55.07
mtlsu	Bryophyta	*Takakia_ceratophylla*	52.97
mtlsu	Marchantiophyta	*Targionia_hypophylla*	53.72
mtlsu	Bryophyta	*Tetraphis_pellucida*	53.91
mtlsu	Gimnosperms	*Zamia_integrifolia*	54.29
mtlsu	Gimnosperms	*Araucaria_araucana*	56.57
nssu	Angiosperms	*Akebia_quinata*	60.78
nssu	Angiosperms	*Alisma_plantago-aqua*	60.58
nssu	Angiosperms	*Amborella_trichopoda*	64.02
nssu	Monilophyta	*Anemia_phyllitidis*	58.97
nssu	Anthocerotophyta	*Anthoceros_agrestis*	57.85
nssu	Angiosperms	*Areca_triandra*	63.32
nssu	Angiosperms	*Asparagus_officinali*	61.03
nssu	Monilophyta	*Asplenium_australasi*	60.46
nssu	Angiosperms	*Austrobaileya_scande*	60.53
nssu	Monilophyta	*Azolla_filiculoides*	59.32
nssu	Zygnematophyta	*Bambusina_borreri*	58.06
nssu	Marchantiophyta	*Bazzania_trilobata*	61.75
nssu	Marchantiophyta	*Blasia_pusilla*	60.62
nssu	Monilophyta	*Blechnum_occidentale*	59.59
nssu	Angiosperms	*Brachypodium_distach*	58.80
nssu	Angiosperms	*Brasenia_schreberi*	61.69
nssu	Bryophyta	*Buxbaumia_aphylla*	58.88
nssu	Monilophyta	*Calochlaena_dubia*	60.18
nssu	Angiosperms	*Carludovica_palmata*	62.47
nssu	Gimnosperms	*Cedrus_deodara*	59.18

nssu	Angiosperms	*Ceratophyllum_echina*	60.48
nssu	Coleochaetophyta	*Chaetosphaeridium_gl*	58.29
nssu	Charophyta	*Chara_braunii*	59.10
nssu	Charophyta	*Chara_fibrosa*	58.71
nssu	Klebsormidiophyta	*Chlorokybus_atmophyt*	56.64
nssu	Zygnematophyta	*Closterium_acerosum*	56.99
nssu	Coleochaetophyta	*Coleochaete_conchata*	56.34
nssu	Coleochaetophyta	*Coleochaete_nitellar*	55.70
nssu	Marchantiophyta	*Conocephalum_conicum*	62.46
nssu	Angiosperms	*Corydalis_saxicola*	61.11
nssu	Angiosperms	*Croomia_pauciflora*	61.31
nssu	Gimnosperms	*Cycas_taitungensis*	57.78
nssu	Zygnematophyta	*Cylindrocystis_brebi*	55.48
nssu	Monilophyta	*Danaea_elliptica*	61.35
nssu	Zygnematophyta	*Desmidium_grevillei*	56.82
nssu	Monilophyta	*Dicksonia_antarctica*	59.62
nssu	Bryophyta	*Dicranum_scoparium*	60.48
nssu	Angiosperms	*Digitalis_purpurea*	61.06
nssu	Angiosperms	*Dioscorea_alata*	62.95
nssu	Bryophyta	*Diphyscium_foliosum*	58.51
nssu	Monilophyta	*Dryopteris_wallichia*	59.64
nssu	Gimnosperms	*Ephedra_fragilis*	59.23
nssu	Monilophyta	*Equisetum_arvense*	59.49
nssu	Bryophyta	*Fissidens_taxifolius*	60.15
nssu	Zygnematophyta	*Genicularia_spirotae*	53.83
nssu	Gimnosperms	*Ginkgo_biloba*	61.75
nssu	Gimnosperms	*Gnetum_indicum*	60.14
nssu	Zygnematophyta	*Gonatozygon_aculeatu*	53.96
nssu	Angiosperms	*Grevillea_robusta*	59.75
nssu	Zygnematophyta	*Haplotaenium_minutum*	56.14
nssu	Klebsormidiophyta	*Hormidiella_attenuat*	59.53
nssu	Angiosperms	*Hortonia_floribunda*	62.70
nssu	Lycopodiophyta	*Huperzia_taxifolia*	59.30
nssu	Bryophyta	*Hypnum_cupressiforme*	59.73
nssu	Klebsormidiophyta	*Interfilum_paradoxum*	58.90
nssu	Klebsormidiophyta	*Klebsormidium_flacci*	58.78
nssu	Angiosperms	*Lactuca_sativa*	59.54
nssu	Charophyta	*Lamprothamnium_macro*	59.12
nssu	Charophyta	*Lychnothamnus_barbat*	59.94
nssu	Lycopodiophyta	*Lycopodiella_inundat*	60.53
nssu	Lycopodiophyta	*Lycopodium_clavatum*	59.83
nssu	Angiosperms	*Magnolia_tripetala*	61.46
nssu	Marchantiophyta	*Marchantia_polymorph*	62.91
nssu	Angiosperms	*Medicago_truncatula*	59.90
nssu	Mesostigmatophyta	*Mesostigma_viride*	60.58
nssu	Charophyta	*Nitella_hyalina*	62.25
nssu	Charophyta	*Nitellopsis_obtusa*	58.99
nssu	Anthocerotophyta	*Nothoceros_giganteus*	58.22
nssu	Anthocerotophyta	*Notothylas_breutelii*	58.39
nssu	Marchantiophyta	*Nowellia_curvifolia*	60.27
nssu	Marchantiophyta	*Odontoschisma_denuda*	60.73
nssu	Angiosperms	*Oenothera_macrocarpa*	58.43
nssu	Marchantiophyta	*Pallavicinia_lyellii*	61.65
nssu	Angiosperms	*Panax_ginseng*	59.56
nssu	Marchantiophyta	*Pellia_epiphylla*	59.27
nssu	Anthocerotophyta	*Phaeoceros_laevis*	58.99

nssu	Anthocerotophyta	*Phymatoceros_phymato*	59.14
nssu	Bryophyta	*Physcomitrella_paten*	58.67
nssu	Gimnosperms	*Pinus_wallichiana*	59.66
nssu	Marchantiophyta	*Plagiochila_adiantoi*	62.63
nssu	Marchantiophyta	*Pleurozia_purpurea*	53.97
nssu	Bryophyta	*Polytrichum_juniperi*	60.09
nssu	Marchantiophyta	*Ptilidium_pulcherrim*	62.12
nssu	Marchantiophyta	*Reboulia_hemisphaeri*	63.50
nssu	Marchantiophyta	*Riccia_fluitans*	63.87
nssu	Angiosperms	*Ricinus_communis*	61.10
nssu	Angiosperms	*Saruma_henryi*	59.23
nssu	Marchantiophyta	*Scapania_nemorea*	61.38
nssu	Marchantiophyta	*Solenostoma_hyalinum*	62.25
nssu	Marchantiophyta	*Sphaerocarpos_donnel*	62.27
nssu	Bryophyta	*Sphagnum_palustre*	62.23
nssu	Bryophyta	*Takakia_ceratophylla*	59.14
nssu	Gimnosperms	*Taxus_cuspidata*	59.44
nssu	Bryophyta	*Tetraphis_pellucida*	59.18
nssu	Angiosperms	*Tofieldia_calyculata*	61.41
nssu	Charophyta	*Tolypella_nidifica*	62.22
nssu	Angiosperms	*Vanilla_africana*	60.63
nssu	Gimnosperms	*Zamia_pumila*	61.29
nssu	Angiosperms	*Zea_mays*	61.65
nssu	Gimnosperms	*Araucaria_araucana*	60.41
cpssu	Monilophyta	*Adiantum_capillus-ve*	65.83
cpssu	Angiosperms	*Alisma_plantago-aqua*	63.53
cpssu	Angiosperms	*Amaranthus_tubercula*	64.94
cpssu	Angiosperms	*Amborella_trichopoda*	64.53
cpssu	Monilophyta	*Anemia_phyllitidis*	65.32
cpssu	Monilophyta	*Angiopteris_evecta*	64.84
cpssu	Anthocerotophyta	*Anthoceros_formosae*	63.07
cpssu	Monilophyta	*Asplenium_nidus*	64.81
cpssu	Bryophyta	*Atrichum_angustatum*	62.81
cpssu	Angiosperms	*Austrobaileya_scande*	64.29
cpssu	Monilophyta	*Azolla_sp*	67.57
cpssu	Marchantiophyta	*Bazzania_trilobata*	58.00
cpssu	Marchantiophyta	*Blasia_pusilla*	58.09
cpssu	Monilophyta	*Blechnum_gibbum*	66.26
cpssu	Monilophyta	*Botrychium_biternatu*	65.94
cpssu	Bryophyta	*Buxbaumia_aphylla*	62.66
cpssu	Angiosperms	*Buxus_sempervirens*	65.04
cpssu	Angiosperms	*Cabomba_sp*	64.74
cpssu	Gimnosperms	*Cedrus_deodara*	64.58
cpssu	Angiosperms	*Ceratophyllum_demers*	64.26
cpssu	Monilophyta	*Ceratopteris_sp*	64.28
cpssu	Charophyta	*Chara_corallina*	58.42
cpssu	Charophyta	*Chara_sp*	58.49
cpssu	Charophyta	*Chara_vulgaris*	58.67
cpssu	Klebsormidiophyta	*Chlorokybus_atmophyt*	61.42
cpssu	Monilophyta	*Cibotium_glaucum*	66.48
cpssu	Angiosperms	*Citrus_sinensis*	65.42
cpssu	Coleochaetophyta	*Coleochaete_nitellar*	59.05
cpssu	Coleochaetophyta	*Coleochaete_orbicula*	59.53
cpssu	Coleochaetophyta	*Coleochaete_scutata*	59.11
cpssu	Gimnosperms	*Cycas_taitungensis*	65.18
cpssu	Monilophyta	*Davallia_fejeensis*	65.17

cpssu	Angiosperms	*Dicentra_sp2*	64.67
cpssu	Bryophyta	*Dicranum_scoparium*	61.91
cpssu	Angiosperms	*Dioscorea_elephantip*	63.83
cpssu	Lycopodiophyta	*Diphasiastrum_digita*	63.95
cpssu	Bryophyta	*Diphyscium_foliosum*	62.65
cpssu	Angiosperms	*Drimys_granadensis*	64.39
cpssu	Monilophyta	*Dryopteris_wallichia*	66.14
cpssu	Klebsormidiophyta	*Entransia_fimbriata*	57.28
cpssu	Monilophyta	*Equisetum_arvense*	65.79
cpssu	Monilophyta	*Equisetum_hyemale*	65.79
cpssu	Bryophyta	*Fissidens_dubius*	62.15
cpssu	Marchantiophyta	*Frullania_dilatata*	58.16
cpssu	Gimnosperms	*Ginkgo_biloba*	65.24
cpssu	Angiosperms	*Glechoma_hederacea*	65.84
cpssu	Gimnosperms	*Gnetum_parvifolium*	59.59
cpssu	Lycopodiophyta	*Huperzia_lucidula*	63.77
cpssu	Bryophyta	*Hypnum_imponens*	61.73
cpssu	Lycopodiophyta	*Isoetes_flaccida*	66.66
cpssu	Angiosperms	*Lactuca_sativa*	63.86
cpssu	Marchantiophyta	*Leiomylia_anomala*	56.65
cpssu	Marchantiophyta	*Lepidogyna_hodgsonia*	59.67
cpssu	Angiosperms	*Liriodendron_tulipif*	64.42
cpssu	Marchantiophyta	*Lophozia_gillmani*	56.64
cpssu	Marchantiophyta	*Marchantia_polymorph*	58.25
cpssu	Marchantiophyta	*Marsupella_emarginat*	57.99
cpssu	Angiosperms	*Medicago_truncatula*	61.88
cpssu	Mesostigmatophyta	*Mesostigma_viride*	58.81
cpssu	Zygnematophyta	*Mesotaenium_caldario*	53.68
cpssu	Angiosperms	*Myristica_yunnanensi*	63.95
cpssu	Angiosperms	*Nandina_domestica*	64.96
cpssu	Angiosperms	*Nelumbo_nucifera*	64.95
cpssu	Marchantiophyta	*Nowellia_curvifolia*	57.12
cpssu	Marchantiophyta	*Odontoschisma_denuda*	57.53
cpssu	Angiosperms	*Oenothera_bienni*	65.24
cpssu	Angiosperms	*Panax_ginseng*	65.17
cpssu	Marchantiophyta	*Pellia_epiphylla*	59.43
cpssu	Anthocerotophyta	*Phaeoceros_laevis*	63.81
cpssu	Angiosperms	*Phoenix_dactylifera*	64.82
cpssu	Bryophyta	*Physcomitrella_paten*	62.69
cpssu	Gimnosperms	*Pinus_pinaster*	64.03
cpssu	Angiosperms	*Piper_nigrum*	64.39
cpssu	Marchantiophyta	*Plagiochila_porelloi*	58.32
cpssu	Gimnosperms	*Podocarpus_macrophyl*	59.70
cpssu	Bryophyta	*Polytrichum_commune*	63.46
cpssu	Marchantiophyta	*Ptilidium_pulcherrim*	60.56
cpssu	Marchantiophyta	*Radula_complanata*	56.90
cpssu	Marchantiophyta	*Reboulia_hemisphaeri*	58.52
cpssu	Marchantiophyta	*Riccia_sorocarpa*	59.00
cpssu	Marchantiophyta	*Ricciocarpos_natans*	59.14
cpssu	Marchantiophyta	*Scapania_nemorea*	58.50
cpssu	Angiosperms	*Schisandra_sphenanth*	64.17
cpssu	Lycopodiophyta	*Selaginella_uncinata*	68.86
cpssu	Angiosperms	*Silene_latifolia*	64.93
cpssu	Marchantiophyta	*Solenostoma_hyalinum*	57.80
cpssu	Angiosperms	*Spathiphyllum_wallis*	63.61
cpssu	Bryophyta	*Sphagnum_capillifoli*	65.79

cpssu	Bryophyta	*Sphagnum_palustre*	65.40
cpssu	Zygnematophyta	*Spirogyra_maxima*	55.77
cpssu	Gimnosperms	*Stangeria_eriopus*	65.18
cpssu	Marchantiophyta	*Symphyogyna_circinat*	60.74
cpssu	Marchantiophyta	*Targionia_hypophylla*	58.99
cpssu	Gimnosperms	*Taxus_cuspidata*	55.43
cpssu	Bryophyta	*Tetraphis_pellucida*	63.23
cpssu	Angiosperms	*Tofieldia_calyculata*	64.42
cpssu	Gimnosperms	*Welwitschia_mirabili*	60.13
cpssu	Gimnosperms	*Zamia_integrifolia*	64.23
cpssu	Gimnosperms	*Araucaria_araucana*	61.32
cpssu	Angiosperms	*Platanus_occidentali*	63.73
cplsu	Gimnosperms	*Abies_homolepis*	59.84
cplsu	Zygnematophyta	*Actinotaenium_crucif*	58.32
cplsu	Monilophyta	*Alsophila_spinulosa*	59.02
cplsu	Angiosperms	*Amborella_trichopoda*	60.76
cplsu	Monilophyta	*Anemia_phyllitidis*	59.13
cplsu	Marchantiophyta	*Aneura_mirabilis*	57.71
cplsu	Monilophyta	*Angiopteris_evecta*	58.00
cplsu	Anthocerotophyta	*Anthoceros_formosae*	57.14
cplsu	Monilophyta	*Asplenium_nidus*	58.91
cplsu	Bryophyta	*Atrichum_angustatum*	56.65
cplsu	Angiosperms	*Austrobaileya_scande*	60.80
cplsu	Monilophyta	*Azolla_sp*	59.59
cplsu	Zygnematophyta	*Bambusina_borreri*	56.43
cplsu	Marchantiophyta	*Bazzania_trilobata*	56.44
cplsu	Marchantiophyta	*Blasia_pusilla*	55.46
cplsu	Monilophyta	*Blechnum_gibbum*	60.18
cplsu	Monilophyta	*Botrychium_dissectum*	59.47
cplsu	Angiosperms	*Brachypodium_distach*	60.24
cplsu	Bryophyta	*Buxbaumia_aphylla*	55.31
cplsu	Angiosperms	*Buxus_sempervirens*	60.91
cplsu	Angiosperms	*Cabomba_sp*	60.84
cplsu	Gimnosperms	*Cedrus_deodara*	59.83
cplsu	Angiosperms	*Ceratophyllum_demers*	61.44
cplsu	Coleochaetophyta	*Chaetosphaeridium_gl*	54.82
cplsu	Charophyta	*Chara_contraria*	53.28
cplsu	Charophyta	*Chara_corallina*	52.99
cplsu	Charophyta	*Chara_vulgaris*	53.28
cplsu	Monilophyta	*Cibotium_sp*	58.53
cplsu	Angiosperms	*Citrus_sinensis*	60.89
cplsu	Coleochaetophyta	*Coleochaete_nitellar*	47.74
cplsu	Coleochaetophyta	*Coleochaete_orbicula*	47.84
cplsu	Marchantiophyta	*Conocephalum_conicum*	55.53
cplsu	Angiosperms	*Conopholis_americana*	56.63
cplsu	Zygnematophyta	*Cosmarium_botrytis*	56.63
cplsu	Zygnematophyta	*Cosmarium_sinostegos*	57.70
cplsu	Gimnosperms	*Cycas_taitungensis*	60.26
cplsu	Zygnematophyta	*Cylindrocystis_brebi*	49.27
cplsu	Monilophyta	*Danaea_elliptica*	57.61
cplsu	Monilophyta	*Davallia_fejeensis*	60.45
cplsu	Anthocerotophyta	*Dendroceros_granulat*	57.49
cplsu	Bryophyta	*Dicranum_scoparium*	56.24
cplsu	Angiosperms	*Dioscorea_alata*	60.99
cplsu	Bryophyta	*Diphyscium_foliosum*	56.31
cplsu	Monilophyta	*Dryopteris_wallichia*	58.64

cplsu	Klebsormidiophyta	*Entransia_fimbriata*	53.14
cplsu	Gimnosperms	*Ephedra_equisetina*	56.79
cplsu	Monilophyta	*Equisetum_arvense*	57.90
cplsu	Monilophyta	*Equisetum_hyemale*	58.17
cplsu	Bryophyta	*Fissidens_dubius*	55.30
cplsu	Marchantiophyta	*Frullania_dilatata*	56.00
cplsu	Gimnosperms	*Ginkgo_biloba*	59.99
cplsu	Zygnematophyta	*Gonatozygon_brebisso*	53.37
cplsu	Bryophyta	*Hypnum_imponens*	55.76
cplsu	Lycopodiophyta	*Isoetes_flaccida*	60.79
cplsu	Angiosperms	*Lactuca_sativa*	60.22
cplsu	Marchantiophyta	*Leiomylia_anomala*	56.11
cplsu	Marchantiophyta	*Lejeunea_cavifolia*	54.95
cplsu	Angiosperms	*Liriodendron_tulipif*	60.94
cplsu	Marchantiophyta	*Marchantia_polymorph*	55.41
cplsu	Marchantiophyta	*Marsupella_emarginat*	55.29
cplsu	Angiosperms	*Medicago_truncatula*	58.69
cplsu	Anthocerotophyta	*Megaceros_tosanus*	58.18
cplsu	Mesostigmatophyta	*Mesostigma_viride*	55.79
cplsu	Zygnematophyta	*Mesotaenium_braunii*	60.36
cplsu	Marchantiophyta	*Metzgeria_conjugata*	56.32
cplsu	Zygnematophyta	*Micrasterias_thomasi*	58.31
cplsu	Charophyta	*Nitella_sp*	51.90
cplsu	Anthocerotophyta	*Notothylas_breutelii*	57.87
cplsu	Angiosperms	*Oenothera_biennis*	60.85
cplsu	Angiosperms	*Panax_ginseng*	60.92
cplsu	Marchantiophyta	*Pellia_neesiana*	57.91
cplsu	Anthocerotophyta	*Phaeoceros_carolinia*	57.76
cplsu	Angiosperms	*Phoenix_dactylifera*	60.46
cplsu	Gimnosperms	*Pinus_koraiensis*	59.37
cplsu	Angiosperms	*Piper_betle*	60.79
cplsu	Marchantiophyta	*Plagiochila_porelloi*	55.65
cplsu	Angiosperms	*Platanus_occidentali*	61.06
cplsu	Gimnosperms	*Podocarpus_macrophyl*	57.22
cplsu	Bryophyta	*Polytrichum_juniperi*	56.36
cplsu	Marchantiophyta	*Reboulia_hemisphaeri*	55.56
cplsu	Marchantiophyta	*Riccia_sorocarpa*	55.64
cplsu	Marchantiophyta	*Ricciocarpos_natans*	56.06
cplsu	Angiosperms	*Ricinus_communis*	56.97
cplsu	Marchantiophyta	*Scapania_nemorea*	55.59
cplsu	Lycopodiophyta	*Selaginella_moellend*	61.77
cplsu	Lycopodiophyta	*Selaginella_uncinata*	62.92
cplsu	Marchantiophyta	*Sphaerocarpos_donnel*	55.34
cplsu	Bryophyta	*Sphagnum_palustre*	58.60
cplsu	Bryophyta	*Sphagnum_rubellum*	58.46
cplsu	Angiosperms	*Spinacia_oleracea*	59.63
cplsu	Gimnosperms	*Stangeria_eriopus*	60.02
cplsu	Zygnematophyta	*Staurastrum_lunatum*	59.29
cplsu	Zygnematophyta	*Staurodesmus_converg*	58.45
cplsu	Bryophyta	*Takakia_lepidozioide*	58.84
cplsu	Bryophyta	*Tetraphis_pellucida*	57.31
cplsu	Angiosperms	*Tofieldia_calyculata*	60.86
cplsu	Angiosperms	*Yucca_schidigera*	60.39
cplsu	Angiosperms	*Zea_mays*	59.75
cplsu	Klebsormidiophyta	*Chlorokybus_atmophyt*	56.38
cplsu	Klebsormidiophyta	*Klebsormidium_flacci*	60.60

Figure S1. Linear regression (1.dashed lines), Phylogenetic Generalized Least Squares (PGLS) method (1. continuous lines) and "crunch" method (2) for the relationships between genomic variables, with all species (black), excluding the species of Chlorophyta (red) and excluding the species of Streptophyta (green): (**A**) The effect of the log transformed number of repeated sequences (NRS), on non-coding genome (%NC); (**B**) Effect of %NC on log transformed Genome length (GL); (**C**) Effect of log(NRS) on GC content (%GC); (**D**) Effect of %NC on %GC; (**E**) Effect of the number of protein-coding genes (NPG) on the log(GL).

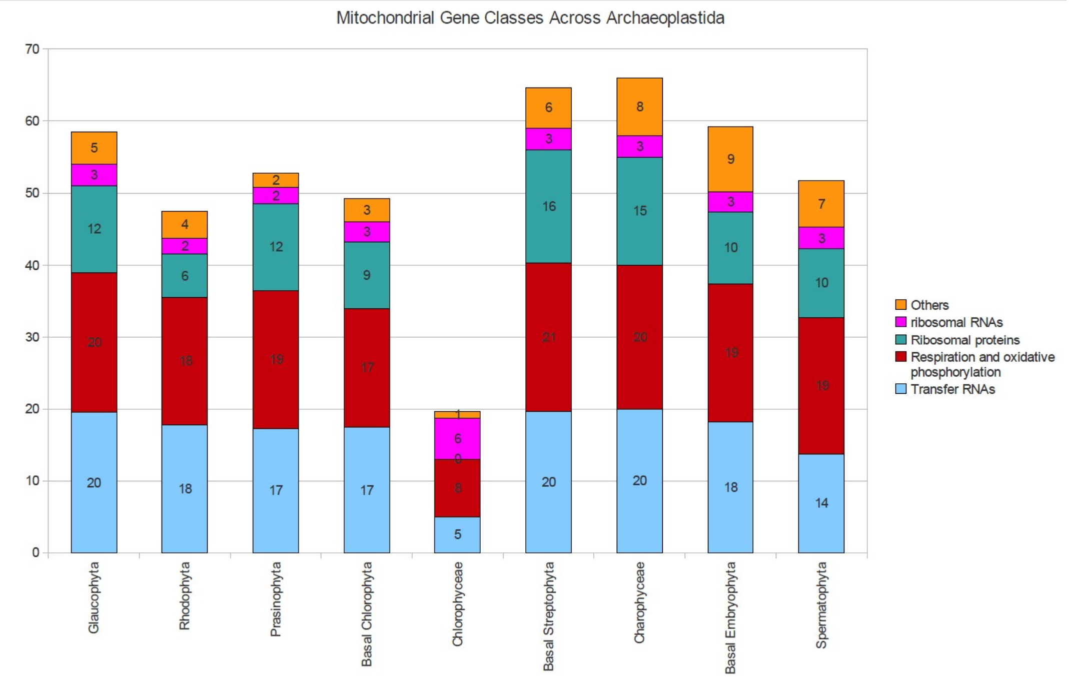

Figure S2. Mitochondrial gene classes across Archaeplastida. Average gene number for each function in each clade.

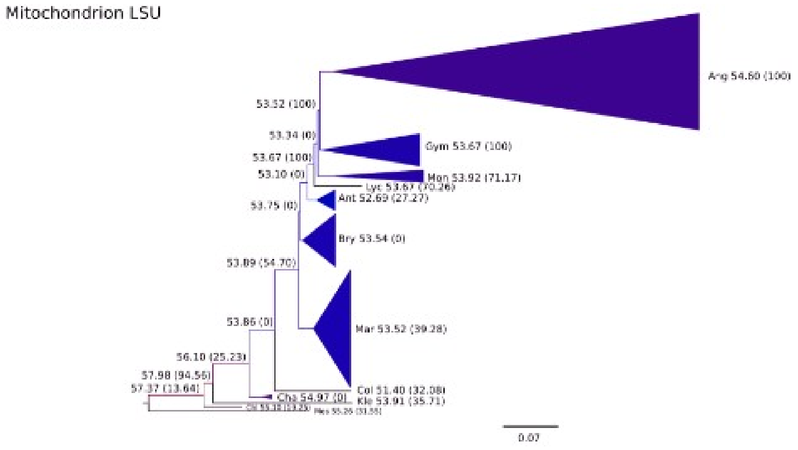

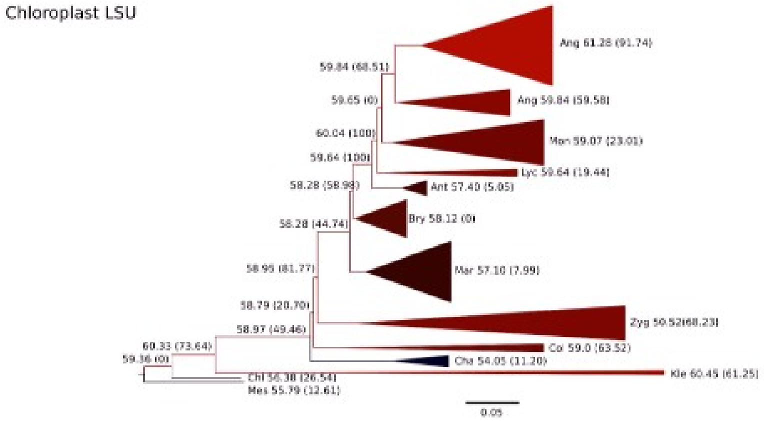

Figure S3. Evolution of GC content and GC* across the phylogeny of Streptophyta: A) Mitochondrial ribosomal LSU; B) Plastidial ribosomal LSU. Values correspond to ancestral GC content at nodes, or GC* (equilibrium GC content) in parentheses. Colors in terminal branches represent average GC content (blue: lowest GC content; red: highest GC content). The color scale is relative to the data set in each tree and is not directly comparable between them. List of species and GC content at terminal branches of each ribosomal subunit is available in Supplementary Table S3.
